# Validity of Klotho, CYR61 and YKL-40 as ideal predictive biomarkers for acute kidney injury: review study: Validade de Klotho, CYR61 e YKL-40 como biomarcadores preditivos ideais para lesão renal aguda: estudo de revisão

**DOI:** 10.1590/1516-3180.2016.0099220516

**Published:** 2016-10-13

**Authors:** Osama Mosa, Milan Skitek, Ales Jerin

**Affiliations:** I PhD. Lecturer of Clinical Biochemistry, Department of Public Health, Health Science College at Al-Leith, Umm Al-Qura University, Saudi Arabia.; II PhD. Professor and Head of Institute of Clinical Chemistry and Biochemistry, Ljubljana University Medical Center, Ljubljana, Slovenia.; III PhD. Associate Professor and Head of Department of Hormones and Tumor Markers, Institute of Clinical Chemistry and Biochemistry, Ljubljana University Medical Center, Ljubljana, Slovenia.

**Keywords:** Acute kidney injury, Thoracic surgery, Proteins, Biomarkers, Review

## Abstract

**CONTEXT AND OBJECTIVE:**

Acute kidney injury (AKI) is still a headache for clinicians and scientists as a possible reason for increased death among intensive care unit (ICU) patients after invasive cardiac surgery. Furthermore, the diagnostic process for AKI using conventional biomarkers is not sufficient to ensure early warning of this condition because of the morbid influence of non-renal factors that definitively delay the time for the prognosis. These imposed limitations have led to significant amounts of research targeted towards identifying novel biomarkers for AKI with a sustained degree of sensitivity and specificity. Here, we reviewed previous studies conducted on the Klotho, CYR61 and YKL-40 biomarkers in relation to AKI.

**DESIGN AND SETTING:**

Review of the literature conducted in the Institute of Clinical Chemistry & Biochemistry, Ljubljana University Medical Center, Slovenia.

**METHODS:**

The literature was searched in PubMed and the Cochrane Library. From the database of this specialty, we selected 17 references that matched our context for detailed analysis and further investigation.

**RESULTS:**

The studies reviewed showed notable differences in their results relating to the diagnostic impact of Klotho, CYR61 and YKL-40 on early prediction of AKI.

**CONCLUSIONS:**

The results regarding the Klotho, CYR61 and YKL-40 biomarkers showed markedly equivocal performance in the previous studies and did not fulfill the expectations that these factors would form valid possible biomarkers for AKI.

## RESUMO

**CONTEXTO E OBJETIVO:**

A lesão renal aguda (LRA) ainda é uma dor de cabeça para os clínicos e cientistas como possível razão para o aumento da mortalidade entre os pacientes de unidade de terapia intensiva (UTI) após cirurgia cardíaca invasiva. Além disso, o processo de diagnóstico para LRA usando biomarcadores convencionais não é suficiente para garantir um alerta precoce desta condição, devido à influência mórbida de fatores não renais que podem retardar o tempo para o prognóstico. Essas limitações geraram quantidades significativas de pesquisas orientadas para identificar novos biomarcadores para LRA com um grau adequado de sensibilidade e especificidade. Revisamos estudos anteriores realizados sobre os biomarcadores Klotho, CYR61, YKL-40 para LRA.

**TIPO DE ESTUDO E LOCAL:**

Revisão da literatura realizada no Instituto de Química Clínica e Bioquímica, Centro Médico da Universidade de Ljubljana, Eslovênia.

**MÉTODOS:**

A literatura foi pesquisada no PubMed e Cochrane Library. A partir da base de dados da especialidade, selecionamos 17 referências que combinavam com o contexto para uma análise detalhada e mais investigação.

**RESULTADOS:**

Os estudos revisados mostraram diferenças notáveis nos resultados sobre o impacto diagnóstico de Klotho, CYR61 e YKL-40 sobre a detecção precoce do LRA.

**CONCLUSÃO:**

Os resultados em relação aos biomarcadores Klotho, CYR61 e YKL-40 mostraram desempenho marcadamente equívoco nos estudos anteriores e não cumpriram as expectativas de que estes fatores constituam possíveis biomarcadores válidos para LRA.

## INTRODUCTION

Acute kidney injury (AKI) is a highly progressive critical problem that often occurs after invasive cardiac surgery using cardiopulmonary bypass (CBP).^1^^,^^2^ It threatens the life of intensive care unit (ICU) hospitalized patients through accompanying irreversible adverse outcomes that ultimately contribute to a 60% increase in mortality rate.^3^ Defining AKI is dependent on measurement of baseline serum creatinine, the traditional biomarker of kidney function, which remains unchanged until a sudden 50% of kidney function is lost.^4^ Moreover, AKI has been found to be strongly affected by dietary status, exercise, protein supplements, corticosteroids, age, gender and muscle mass.^5^^,^^6^ Therefore, there is an urgent need for novel biomarkers to predict and diagnose AKI at its earlier stages, so as to prevent complications and potentiate therapeutic approaches.

### Classification of AKI

The Acute Dialysis Quality Initiative Group (ADQI) meeting in 2004 gave rise to a new regular criterion for analyzing kidney function, termed Risk Injury Failure Loss of function and End stage (RIFLE).^7^^,^^8^ RIFLE was dependent on serum creatinine (SCr) or urinary output (UO) measurements to determine the prognostic severity of deterioration of kidney function, classified into three stages.^8^ Many studies mentioned that the usefulness of RIFLE was affected by the following substantial limitations: [1] calculation of the SCr baseline using the Modification of Diet in Renal Disease (MDRD) equation showed high specificity for chronic kidney disease (CKD) but not AKI; [2] SCr was directly influenced by nonspecific factors and hence was unreliable; [3] using UO was a good alternative for SCr, but it was affected by diuretics and could only be measured by using a bladder catheter in an ICU and not among long-stay hospitalized patients; and [4] SCr was considered to be a marker for renal function, not kidney injury.^9^

Subsequently, a modified standard was published in 2007 under the name "Acute Kidney Injury Network (AKIN)", with the aim of closing gaps generated by RIFLE. AKIN used two values of SCr within two days instead of baseline SCr, regardless of glomerular filtration rate (GFR) changes. According to AKIN, stage 3 AKI was confirmed when the duration of increased SCr levels did not exceed 48 h and the patient required renal replacement therapy (RRT).^10^

The failure of both RIFLE and AKIN to fulfill precise prognostic stratification of AKI severity and to provide a unified definition of AKI was the reason for establishing the Kidney Disease Improving Global Outcomes (KDIGO) guidelines. These novel criteria suggested that AKI should be defined by SCr levels that reached 26.4 µmol/l within 48 h or increased to a level 1.5 times higher than the baseline level within 7 days, which provides a sufficient timeframe for AKI diagnosis.^11^ The differences between all the diagnostic criteria are summarized in Table 1.

**Table 1:**
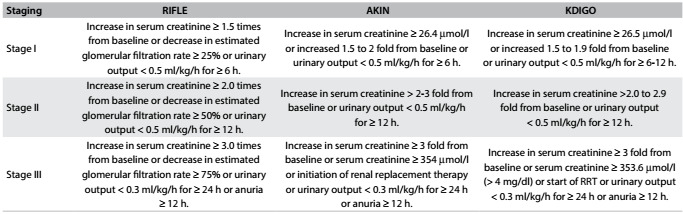
Differences between the guidelines "risk injury failure loss of function and end stage" (RIFLE), "acute kidney injury network" (AKIN) and "kidney disease improving global outcomes" (KDIGO) for diagnosing acute kidney injury (AKI)

### Epidemiology of AKI

The AKI incidence rate worldwide has remained imprecise because of the small number of case report studies, gaps in the data collected from patients and differences in definitions of AKI between developed and developing countries.^12^^,^^13^^,^^14^ Recent studies conducted in the USA and Spain showed incidences of approximately 23.8 cases per 1000 discharges and 209 cases per million, respectively.^15^^,^^16^ A recent population-based study conducted in the UK reported high incidence of AKI, of 1811 cases per million in 2003.^17^ A report from Kuwait indicated an incidence of 4.1 cases per 100,000 population per year.^18^ In addition, the annual incidences for AKI in Brazil and northern India were 7.9 and 6.4 cases per 1000 admissions.^19^^,^^20^ Notably, the mortality rates in developed countries were found to be lower than those in developing countries, where young adults and children were very badly affected.^21^

### Prospective biomarkers

#### Klotho

Klotho (KL) is a novel phosphatonin encoded by the anti-aging KL gene located on chromosome 13q12 as an inactive single-pass transmembrane protein.^22^ Upon activation through action by membrane bound-secretases like ADAM10 and ADAM17, driven by insulin, the extracellular domain is cleaved and its serum, urine and cerebrospinal fluid levels become elevated.^23^ This ectodomain was termed a soluble Klotho, which would possibly bind directly with FGFR and tend to form an active complex exhibiting high affinity against FGF,^24^ thereby alleviating oxidative stress through suppression of growth factors and stimulation of calcium ion channels (TRPV5 and TRPV6)^23^ and potassium channels (ROMK)^25^ but not sodium-phosphate cotransporters.^26^ Meanwhile, the remaining membrane Klotho would function as a coreceptor for bone regulatory hormone FGF23.^27^ Normally, Klotho shows greater expression in distal rather than proximal convoluted tubules in the kidneys, and in the choroid plexus of the brain rather than in the heart and parathyroid gland.^28^

The pathological importance of Klotho emerged through studies on animal models for AKI that had previously undergone ischemic reperfusion injury (IRI) or unilateral urethral obstruction (UUO). Thus, a transient reduction in renal Klotho mRNA expression was shown in response to renal tubular injury.^29^^,^^30^ Other studies on Klotho applied to humans have demonstrated that the urinary and plasma levels of Klotho in patients with AKI are notably lower than in healthy individuals.^29^ From these observations, it has been proposed that Klotho has a role in exacerbating renal damage and has potential as a likely biomarker for AKI.

#### Cysteine-rich protein 61 (CYR61)

CYR61 is a cysteine-rich matricellular protein encoded by the CYR61 gene located on chromosome 1p22.3. It is intercalated with various integrins and heparin sulfate proteoglycans and is associated with extracellular matrix formation, cell adhesion, proliferation, differentiation, angiogenesis, apoptosis and inflammation due to its biochemical features, which resemble Wnt-1 proto-oncogene, and its number of growth factors.^31^ Additionally, renal CYR61 mRNA and protein expression, along with urinary levels, have been found to increase in IRI animal models that suffered from significant hypoxia, despite being indistinguishable at renal levels in normal tissues.^32^^,^^33^ This result provides encouragement to study CYR61 levels in humans, in order to elucidate its preventive and/or predictive role against AKI.

#### Chitinase-3-like protein 1 (YKL-40)

Chitinase-3-like protein 1 (CHI3L1) or YKL-40 is a 40 kDa glycoprotein^34^ that is expressed from the CHI3L1 gene located on chromosome 1q31-q32.^35^ It is considered to be a member of the family of 18 glycoside hydrolases that encompasses chitinases but without any enzymatic activity. It is secreted by various cell types, including macrophages, chondrocytes and some types of cancer cells.^34^ Furthermore, Johansen et al. revealed that YKL-40 increased inflammation through activation of the innate immune response and regulation of tissue remolding.^35^ In addition, Maddens et al. collected urine samples from mice that presented sepsis two days after intrauterine injection of *E. coli* , and from human patients with sepsis. They showed similar quantitative increases in comparison with controls without AKI.^36^ Therefore, studies on YKL-40 remain a prerequisite for understanding the pathophysiology of AKI.

## OBJECTIVE

The objective of the current review was to focus on the suitability and validity of Klotho, CYR61 and YKL-40 as ideal predictive biomarkers for acute kidney injury.

## METHODS

We conducted a comprehensive systematic search by using the main known databases: PubMed, SCOPUS, SciELO, Lilacs, ScienceDirect and Google Scholar. The MeSH search terms included: (''Klotho and Acute Kidney Injury''), (''CYR61 and Acute Kidney Injury'') AND (Chitinase-3-like protein 1 and Acute Kidney Injury''). The search strategy was designed for the PubMed database and was altered as needed for use in other databases. Our last search was finished in January 2016. References were written in the English language. The inclusion criterion was that all research articles, review articles and observational studies included needed to match our context, i.e. "the propensity of CYR61, Klotho and YKL-40 to be novel biomarkers for AKI". Additionally, we excluded papers that investigated these biomarkers in relation to chronic kidney disease (CKD) and other diseases as well as AKI.

## RESULTS

Our search revealed a total of 2917 references. From the title and abstract, while omitting review articles, case reports and similar results, the number of papers was reduced to 17, which included seven relating to the biomarker YKL-40, three relating to CYR61 and seven relating to Klotho (Table 2). Briefly, we summarized the main results and recommendations for each study in Table 3.^29^^,^^30^^,^^32^^,^^36^^,^^37^^,^^38^^,^^39^^,^^40^^,^^41^^,^^42^^,^^43^^,^^44^^,^^45^^,^^46^^,^^47^^,^^48^^,^^49^ Finally, a synopsis of the biomarkers studied, showing general descriptions, functions and techniques used for measurements, was produced as Table 4.

**Table 2:**
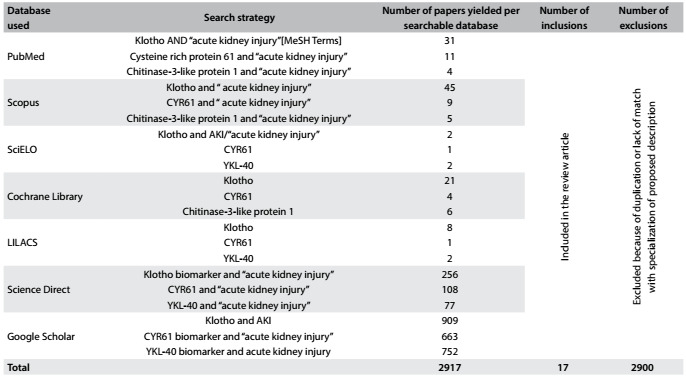
Outlines of the search strategies used for each database

**Table 3:** Summary of characteristics and main results of the 17 previous studies included in this review

Serials	Author/year	Study design	Purpose of the study	Results and recommendations
1	De Loor et al.^37^	Pilot study	To evaluate whether urinary Chitinase 3-like protein 1 (YKL-40) can predict AKI stage ≥ 2 in ICU patients compared with NGAL.	The concentration of UCHI3L1 within 12 hours of AKI stage ≥ 2 was increased with good performance on AUC-ROC curve (0.792, 95% CI), similar to UNGAL AUC-ROC (0.748, 95% CI), and after 24 h, UCHI3L1 showed AUC-ROC twice as high (95% CI: 1.3-3.1) as controls.
2	Huen et al.^38^	Review	Focus on future phenotyping of AKI regarding NGAL and YKL-40.	NGAL and YKL-40 are important novel biomarkers involved in moderate renal tubular protection after AKI.
3	Schmidt et al.^39^	Cohort (comparative) study	To evaluate the role of urinary and blood levels of YKL-40 in allografts after renal transplantation.	Urinary YKL-40 increased early on, within 18 h after surgery (131.3 ± 155.2), with AUC 0.86 ± 0.07; blood YKL-40 retarded to 1 day after surgery (623 ± 285.9), with AUC 0.59 ± 0.08
4	Hall et al.^40^	Observational cohort study	To measure YKL-40 levels in the urine of clinically hospitalized AKI patients.	Urinary YKL-40 levels were detectable (≥ 5 ng/ml) within 1 h and gave better prognostic value (P = 0.04) with NGAL.
5	Tatar et al.^41^	Cohort study	To define relationship between YKL-40 and proteinuria in renal transplant recipients.	Mean serum YKL-40 and proteinuria levels were 66 ± 46 ng/ml and 0.77 ± 1.15 g/day respectively without any apparent correlation.
6	Maddens et al.^36^	Clinical and experimental study	Measurement of urinary and plasma levels of Chitinase 3-like protein 1 and -3 in mice and patients with and without septic AKI.	Urinary CHI3L1 higher in septic-AKI patients than in non-AKI (P < 0.05), but in septic-AKI mice models, CHI3L1 and -3 were found to be high.
7	Malyszko et al.^42^	Review article	Illustration of candidate biomarkers in cases of delayed graft function as a form of acute kidney injury.	Elevated YKL-40 in both urine and serum levels of patients with DGF, 2 days after transplantation.
8	Muramatsu et al.^32^	Experimental study	To test CYR61 in the urinary levels of mice and rats after immediate renal ischemic reperfusion injury.	CYR61 protein increased first within 1 h and appeared in urine 3-6 h after ischemic renal injury.
9	Lai et al.^43^	Experimental study	To investigate the role of CYR61 after unilateral IRI in mice.	CYR61 was significantly induced at renal and urinary levels after IRI.
10	Xu et al.^44^	Experimental study	To indicate CYR61 expression in renal cell lines under hypoxia	Enhanced expression of renal CYR61 in response to hypoxic ischemic injury.
11	Kim et al.^45^	Cohort study	To determine possible influence of AKI on serum and urinary levels of Klotho, S100A8/A9 and NGAL	Urinary Klotho levels were 13.21 ± 17.32 versus 72.97 ± 17.96 pg/ml (P = 0.002) in pre-renal and intrinsic AKI respectively.
12	Torregrosa et al.^46^	Cohort study	Assessment of urinary Klotho levels in patients after cardiac surgery or coronary angiography.	Klotho levels did not behave as a good early biomarker of AKI.
13	Castellano et al.^47^	Experimental study	To investigate whether or not complement components affect Klotho levels after IRI.	Complement activation result in remarkable decline in renal Klotho levels, 24 h after IRI.
14	Liu et al.^48^	Case-control study	To evaluate serum Klotho levels at different time intervals after cardiac surgery.	Serum Klotho levels were 101.97 ± 16.93 versus 121.64 ± 19.87 (P < 0.01) in AKI and non-AKI group respectively at 0 h and continued until 4 h. After 3 days, serum Klotho values were 120.50 ± 13.17 versus 128.67 ± 18.84.
15	Seo et al.^49^	Retrospective cohort study	Assessment of renal Klotho levels in human samples instead of animal models.	Renal Klotho levels were significant reduced with AKI severity.
16	Hu et al.^29^	Experimental and case-control study	To estimate Klotho at urinary and plasma levels, investigating probable protective ability.	Urinary Klotho values (pmoles/l) were 2.52 ± 0.76 in AKI versus 20.66 ± 1.81 in non-AKI, with P < 0.01.
17	Sugiura et al.^30^	Experimental study	To explain the physiological relevance of renal Klotho after IRI in rats.	Renal Klotho levels were significantly reduced in IRI rats, 24 h after ischemia.

**Table 4:**
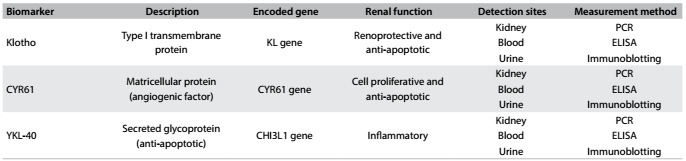
Description of biomarkers, their functions and measurement methods

PCR = polymerase chain reaction; ELISA = enzyme-linked immunosorbent assay.

## DISCUSSION

In this review article, we discuss the propensity of some novel biomarkers for early detection of AKI. Traditional biomarkers have been proven to be unable to satisfactorily distinguish AKI during the first 24 hours before kidney function is disrupted. This is certain to delay the diagnostic process and gives rise to the possibility that the patient's condition will worsen. Despite the paucity of studies on biomarkers and AKI (for reasons mentioned earlier), we conducted a comprehensive review of the literature encompassing all papers relating to our context, focusing on all the results.

Recent papers have inferred that reduced levels of Klotho correlated with emergence of soft tissue calcifications, cardiovascular diseases, senescence, cancers, chronic hypertension, osteoporosis, renal failure, diabetes mellitus, oxidative stress and uremic parathyroid hyperplasia.^50^^,^^51^^,^^52^ Furthermore, Hu et al. observed that Klotho levels in both plasma and urine declined immediately in AKI animal models and were detectable within 3 h after injury. This change preceded any changes in serum creatinine by 1 day and plasma NGAL by 5 h, thus suggesting that Klotho may be an early biomarker for renal parenchymal injury.^29^^,^^53^ In the same manner, Kim et al. demonstrated that there were lower urinary Klotho levels in patients with pre-renal AKI than those with intrinsic AKI, and that this was not accompanied by any change in NGAL at the serum and urinary levels.^45^

Sugiura et al. indicated that renal Klotho levels in rats started to fall on the first day and completely returned to normal within 10 days.^30^ On the other hand, Seo et al. studied human subjects and showed that renal Klotho levels were reduced, compared with high serum creatinine levels, according to AKI severity.^49^ Likewise, Castellano et al. observed that Klotho levels were significantly increased in renal biopsies on cadaveric donors before transplantation and markedly reduced in patients with delayed graft function (DGF), in comparison with patients with early graft function. Furthermore, serum Klotho levels showed a significant decrease in DGF patients two years after transplantation, thus suggesting that the complement component has a modulatory role through activation of the nuclear factor kappa B (NF-kB) signaling pathway.^47^

A clinical study on urinary Klotho levels found that these were lower in AKI patients than in healthy individuals and recommended that this should be a candidate biomarker for AKI.^29^ Surprisingly, Torregrosa et al. concluded that there was no difference in urinary Klotho levels measured by means of the ELISA (enzyme-linked immunosorbent assay) technique between AKI and non-AKI patients after cardiac surgery or coronary angiography, thus dismissing the possibility that Klotho would be a sensitive AKI biomarker.^46^ Recently, Liu et al. showed that there was a notable immediate decline in serum Klotho levels in AKI patients compared with non-AKI (101 ± 16.93 versus 121.64 ± 19.87) after cardiac valve replacement surgery, although the preoperative levels had been steady and close together without any significant difference. Subsequently, 24 hours after the operation, the levels exhibited stepwise recovery towards the preoperative (baseline) levels. This observation indicated that serum Klotho might be a sensitive biomarker limited to a short time after surgery. An emerging suggestion to use the SCr/KL ratio instead of serum creatinine or Klotho alone could improve their diagnostic sensitivity for AKI at later times.^48^

Studies on Klotho were found to exhibit a variety of problematic issues: almost all the studies related to animal models rather than humans, with a narrow scale; there were unexplained variations between comparable studies; the mechanism of Klotho in AKI remains unknown, the behavior of Klotho in animal models differed from its behavior in humans; there was a lack of knowledge of ideal Klotho timing and normal cutoff ranges; and the urinary and plasma levels of Klotho were not indicative for renal Klotho, which might suggest that confounding factors and discrepancies in laboratory methodologies were present.

According to Vaidya and Muramatsu et al., CYR-61 was rapidly stimulated in the proximal renal tubules and was excreted in urine within 3-6 h after bilateral renal ischemic injury in rats. Its peak was within 6-9 h and it declined after 24 h.^32^^,^^54^ Consequently, urinary CYR61 might act as an acceptable biomarker and screening tool for AKI, with follow-up in both preclinical and clinical studies.^32^^,^^55^ Moreover, Lai et al. conducted experiments on mice that proved that proinflammatory TGF-β enhanced renal CYR61 in mRNA and protein levels within 10 days after occurrences of unilateral ureteral obstruction (UUO).^56^ Subsequently, CYR61 gave rise to inflammatory sequelae through activation of monocyte chemoattractant protein-1 (MCP-1), thereby leading to monocyte chemotaxis and macrophage infiltration.^57^ This evidence revealed that inhibition of CYR61 could prevent adverse consequences that would contribute towards irreversible AKI-CKD transition, through postponing inflammation, tubulointerstitial fibrosis and apoptosis.^43^ Furthermore, Xu et al. conducted experiments on renal cell lines under conditions of hypoxia and found that CYR61 expression prevented apoptosis through phosphorylation of BAD, which released anti-apoptotic factors (bcl-2, bcl-xl) and enhanced cell proliferation through activation of the Akt and ERK signaling pathways.^44^

Other previous papers investigating CYR61 expression found that it was induced by several growth factors, exposure to UV irradiation,^58^ hypoxic conditions, vigorous exercise,^59^ bacterial infections^60^ and viral infections.^61^ Likewise, Pendurthi et al. mentioned that clotting factor VIIa (FVIIa) and thrombin triggered CYR61 redundancy, forced through blood coagulation.^62^ This observation matched with Hviid et al., who indicated that CYR61 levels increased at sites of surgical wound closure and that CYR61 was absent from systemic blood, which might explain the mediatory role of platelets in accumulations of CYR61 at sites of tissue injury in AKI patients.^63^

The diagnostic capacity of urinary CYR61 as a biomarker might be blocked through: 1) its poor specificity, since it is normally abundant under both physiological and pathological conditions; 2) its rapid decline over time in spite of AKI progression; 3) the insensitivity of the immunoblotting technique used in quantification in urine; and 4) the fact that most studies were conducted on animal models because of difficulty in obtaining samples from human patients without prolonged routine registry for clinical trials in accordance with the World Health Organization (WHO) requirements and without prior patient approval.

Hall et al. showed that increased levels of urinary YKL-40 of up to 5 ng/ml were moderately correlated with AKI progression and/or mortality among patients. Moreover, apparent increases in YKL-40 levels in urine were observed in cases of kidney transplantation among patients hospitalized within 24 hours of developing AKI.^40^ Further proof was presented by Maddens et al., showing that urinary levels of YKL-40 were elevated in septic AKI patients. Taken together, YKL-40 with the best renal troponins (NGAL) might improve stratification of the risk of AKI among patients without any indications of primary renal damage and strengthen early prediction of sepsis-induced AKI.^36^^,^^38^

Another study by Schmidt and Malyszko et al. reported that urinary YKL-40 was better than serum YKL-40 levels for distinguishing between delayed graft function and slow or immediate graft function, within 3 days after kidney transplantation. Delayed graft function produces greater severity of ischemic kidney injury, while the damage from other types tends to become repaired.^39^^,^^42^ Synergistically, Hall et al. recommended that urinary YKL-40 could be used as an accurate and reliable biomarker to identify patients at risk of AKI following transplantation, rather than urinary or plasma NGAL.^40^ Conversely, a pilot study by De Loor et al. demonstrated that the urinary concentrations of YKL-40 and NGAL in ICU patients with AKI stage ≥ 2 measured within 12 h or 24 h exhibited higher convergent diagnostic performance than did serum YKL-40, which did not show any predictive power against AKI.^37^ Moreover, Tatar et al. concluded that high levels of serum YKL-40 was accompanied by increased CRP and proteinuria levels in kidney transplant recipients, thus indicating its inflammatory role.^41^ Although YKL-40 showed many important benefits, the pathophysiological mechanism that leads to its expression in cases of AKI remains uncertain and validated cutoffs remain largely absent.

## CONCLUSION

The results regarding the Klotho, CYR61 and YKL-40 biomarkers showed markedly equivocal performance in the previous studies and did not fulfill the expectations that these factors would form valid possible biomarkers for AKI.
